# Structure-Based Approaches for Protein–Protein Interaction Prediction Using Machine Learning and Deep Learning

**DOI:** 10.3390/biom15010141

**Published:** 2025-01-17

**Authors:** Despoina P. Kiouri, Georgios C. Batsis, Christos T. Chasapis

**Affiliations:** 1 Institute of Chemical Biology, National Hellenic Research Foundation, 11635 Athens, Greece; despoina.kiouri.99@gmail.com (D.P.K.); georgebatsis95@gmail.com (G.C.B.); 2 Laboratory of Organic Chemistry, Department of Chemistry, National and Kapodistrian University of Athens, 15772 Athens, Greece

**Keywords:** Protein–Protein Interactions, machine learning, deep learning, proteomics, structure representations

## Abstract

Protein–Protein Interaction (PPI) prediction plays a pivotal role in understanding cellular processes and uncovering molecular mechanisms underlying health and disease. Structure-based PPI prediction has emerged as a robust alternative to sequence-based methods, offering greater biological accuracy by integrating three-dimensional spatial and biochemical features. This work summarizes the recent advances in computational approaches leveraging protein structure information for PPI prediction, focusing on machine learning (ML) and deep learning (DL) techniques. These methods not only improve predictive accuracy but also provide insights into functional sites, such as binding and catalytic residues. However, challenges such as limited high-resolution structural data and the need for effective negative sampling persist. Through the integration of experimental and computational tools, structure-based prediction paves the way for comprehensive proteomic network analysis, holding promise for advancements in drug discovery, biomarker identification, and personalized medicine. Future directions include enhancing scalability and dataset reliability to expand these approaches across diverse proteomes.

## 1. Introduction

Proteins are biopolymers of amino acids arranged in polypeptide chains that, along with nucleic acids, lipids and glycans, make up the four fundamental macromolecular components of cells [1]. These versatile macromolecules essentially participate in all cellular processes, such as replication and transcription of DNA, translation, modification and secretion of all proteins as well as the circulation of information and materials in and out of cells [2]. Apart from serving as building blocks and providing structural integrity to the cell, they also participate in biochemical reactions as enzymes, regulate growth, repair and reproduction as hormones, and initiate cell death [3]. Nevertheless, macromolecules are not functional as separate units in the cells, but rather they form complicated interaction networks with one another as well as different macromolecule types. In the case of proteins, the term ‘protein interaction’ entails both physical and functional interactions as well as protein complex formation (transient and stable) [4]. Since proteins play a key role in a multitude of biological processes, their interactions are critical in regulating molecular and cellular mechanisms, influencing both health and disease [4]. To holistically study protein interactions and their synergistic effect, protein–protein interaction (PPI) data can be utilized on a broader scale, and thus they are mapped to interaction networks that are either based on physical or functional associations. Despite being incomplete, the systematic study of protein interaction networks has proven essential for understanding the link between network structure and function, discovering new protein functions, identifying coherent functional modules, and recognizing conserved molecular interaction patterns [4]. To further enrich these networks, a variety of experimental methods have been developed to determine the interactions between proteins. The most important biophysical methods that are used in protein interaction determination tasks are Fluorescence polarization (FP), Surface plasmon resonance (SPR), Nuclear magnetic resonance (NMR), Circular dichroism (CD), Static and dynamic light scattering (SLS/DLS), Analytical ultra-centrifugation and Isothermal titration calorimetry (ITC), that have shown improved performance in studying the hydrodynamic and thermodynamic aspects of PPIs due to advancements in instrumentation [5]. As far as biochemical methods are concerned, Fluorescence and bioluminescence resonance energy transfer (FRET and BRET), bead-based proximity assays (amplified luminescent proximity homogeneous assay (Alpha) Screen and AlphaLISA), Protein-fragment complementation assay (PCA), Affinity chromatography and Cross-linking, that are often combined with mass spectrometry (MS) techniques have been widely applied in the identification of protein binding partners. Furthermore, high-throughput genetic methods like Phage display, Yeast two-hybrid (Y2H) system and other two-hybrid systems as well as protein microarrays have resulted from the genomics revolution, enabling large-scale PPI identification and mapping.

Despite the plethora of methods for PPI identification that have been developed, the experimental verification of all the possible protein interactions is not possible due to the innate limitations of the methods (such as cost and time) and the specific microenvironment of some proteins that make their experimental detection almost impossible (i.e., pH, temperature, etc.) [6]. Nevertheless, the most important factor is the fact that the experimental verification of every possible protein interaction of the proteome of an organism is virtually impossible [7]. To address this issue, in the last few years, many in silico methods for PPI identification have been developed.

In the early days, protein interaction prediction was based on the interaction of the protein’s domains, the parts of a protein that have the ability to fold, function, and evolve independently [8]. The interaction between the domains were inferred from known PPIs, using the assumption that two proteins are interacting if they had two interacting domains. The domain–domain interaction (DDI) prediction was initially based on statistical techniques like the Association Method, and Maximum Likelihood Estimation [9]. Later, optimization algorithms such as Linear Programming [10] and Genetic Algorithm [11] were used to determine the smallest number of DDIs that satisfy a given PPI network (PPIN). Afterward, PPI prediction was performed with Machine Learning (ML)-based techniques using domain knowledge like Random Forest (RF) [12], and recently Graph Theory has been developed as a modern alternative for PPI predictions of this type [13].

Furthermore, docking methods including HADDOCK [14], ClusPro [15], ZDOCK [16], LightDock [17], and InterEvDock [18], were utilized to predict PPIs by physically aligning two proteins to identify a possible binding site. Template-based computational approaches for PPI prediction and structural model construction take advantage of the accumulated sequence and structure knowledge of known PPIs [19,20]. Computational methods for PPI prediction have advanced significantly in recent years due to the quick development of artificial intelligence (AI) algorithms. Sequence-based [21,22,23] and structure-based [24,25,26] are the two main groups into which these AI techniques fall.

At first, PPI prediction was based on the primary protein sequence as well as some physicochemical properties, including hydrophilicity, charge, surface tension, using ML algorithms, such as Support Vector Machine (SVM) [27]. Other methods were oriented towards sequence-signatures (e.g., domains, motifs, etc.) instead of entire sequences in order to identify over-represented sequence–signature pairs in PPIs and perform predictions using statistically-based ML algorithms [28]. Later on, some methodologies that only take advantage of the primary protein sequence and the distribution of amino acids in the sequence were developed, such as the method of You et al. [29] that utilized a novel Multi-scale local descriptor (MLD) feature representation scheme for different lengths of amino acid sequences and an RF Classifier. Some recent methodologies deploy numerous physicochemical characteristics along with the protein sequence with more advanced and fast ML methods, such as Gradient Boosting Decision Tree (GBDT) [30]. Finally, more intricate ML algorithms such as Ensemble Extreme Learning Machine (E-ELM) combined with Principal Component Analysis (PCA) for feature reduction have also been employed in order to utilize more complex sequence-based features, like local sequence patterns, compositional and physicochemical properties as well as global sequence correlations [31]. Deep Learning (DL) methods have also emerged as potent and versatile tools for PPI prediction based on sequence. Sun et al. developed a methodology that uses both Autocovariance (AC) and Conjoint Triad (CT) for differential feature extraction from protein sequences and performs the PPI prediction task via a Stacked Autoencoder (SAE) with a softmax classifier [21]. Currently, Convolutional Neural Networks (CNNs) are widely used for both the extraction of sequence-derived features and the prediction task itself [32].

Even though sequence-based methods have been widely used for PPI prediction tasks since they require far fewer computational resources and can be applied to a greater subset of proteins (i.e., many proteins have available sequence but not structural information) compared with structure-based methods, they cannot compete with the accuracy that structural methods provide [33]. In this work, we discuss the recent advances in protein interaction prediction based on structure, that provide greater biological accuracy, as well as some innovative protein-interaction type and protein-binding site prediction methods.

## 2. PPI and Protein Data

### 2.1. Protein Interaction Information

Over the past years, the number of PPIs has increased substantially, and thus databases that host information about PPIs in a multitude of organisms have emerged. Currently, there are various recognized databases that provide PPIs to design networks, such as DIP, MINT, Biogrid, IntAct and STRING. These databases’ PPIs can be utilized both to train the prediction algorithms used in PPI prediction and to assess the accuracy of the prediction findings, as the available protein interactions have been experimentally verified. Nevertheless, some of those databases also contain predicted protein interactions and thus to perform accurate predictions the predicted interactions should probably not be included. For the correct training of prediction algorithms, “negative” PPI datasets, i.e., datasets that contain proteins that do not interact are needed. The Negatome database contains about 2000 negative interactions both from manual literature curation and 3D protein complexes [34]. However, the number of interactions in Negatome is limited and it is also evident that there are far more negative interactions that have been observed than those that have been published. Negative PPI datasets are often generated using subcellular localization or random sampling, but these methods can introduce biases, overestimate prediction accuracy, or result in unbalanced datasets, especially when studying specific biological contexts [35].

### 2.2. Biological Features of Proteins for PPI Prediction

To perform PPI prediction, biological information in the form of “features”, i.e., individual measurable properties, also needs to be integrated into computational models. In structure-based modeling, the key feature used in PPI predictions is protein structure and thus it is either retrieved from the Protein Data Bank (PDB) [36,37], that contains experimentally supported 3D structures of proteins, or from the AlphaFold Database (last update: September 2024), that contains highly accurate predictions of protein structures [38,39]. From the UniProt database Release 2024_06, a number of supplementary features can be extracted ranging from the protein sequence, post translational modifications events, expression, subcellular location, variants and implication in disease, family and domain information, as well as Gene Ontology (GO) annotations [40]. The PPI interaction databases and meta-databases as well as protein feature databases that are mostly used are presented in Table 1.

## 3. PPI Prediction Methods

**Figure 1:**
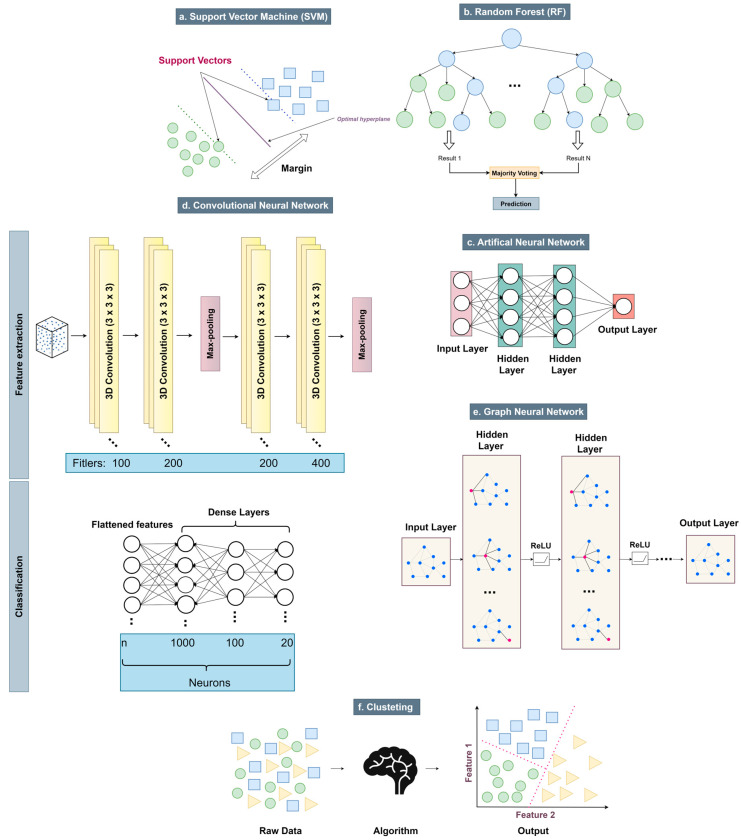
Overview of computational methodologies for structure-based Protein–Protein Interaction prediction: (**a**). Support Vector Machine (SVM): a supervised learning algorithm that classifies interacting and non-interacting protein pairs by constructing an optimal hyperplane, (**b**). Random Forest (RF): an ensemble method employing decision trees to predict PPIs through majority voting mechanisms, (**c**). Artificial Neural Network (ANN): a multilayer perceptron architecture that learns patterns in PPI features, (**d**). Convolutional Neural Network (CNN): a DL model utilizing 3D convolutional layers to process spatial protein features and extract hierarchical representations and (**e**). Graph Neural Network (GNN): a graph-based DL framework that models protein structure as nodes and edges, enabling PPI prediction through graph embeddings, (**f**). Clustering Algorithm: ML techniques used to organize similar data points into clusters according to a distance or similarity metric.

The prediction of PPIs through structure-based computational methods has witnessed significant advancements due to the integration of ML and DL techniques. Figure 1 presents an overview of the key methodologies employed in this domain, ranging from classical ML approaches to advanced DL architectures, each tailored to address specific challenges in PPI prediction. SVMs (Figure 1a), one of the earliest ML methods utilized, operates by defining a hyperplane that separates interacting from non-interacting protein pairs based on input features such as surface properties or physicochemical characteristics. RF classifiers (Figure 1b), which construct ensembles of decision trees, improve prediction accuracy by aggregating outputs through majority voting, making them robust to feature variability. These classical methods rely on hand-crafted features extracted from protein structures and are computationally efficient for medium-scale datasets. The advent of DL methods has revolutionized PPI prediction, enabling the automatic extraction of complex patterns from protein structures. Artificial Neural Networks (ANNs) (Figure 1c) employ multilayer perceptrons to capture non-linear relationships in protein interaction data. CNNs (Figure 1d) extend this capability by processing 3D structural representations of proteins, enabling the identification of spatially relevant features through convolutional operations and pooling. Graph Neural Networks (GNNs) (Figure 1e) model proteins as graphs, where amino acids are represented as nodes and their spatial proximities as edges, thus providing a framework for capturing the topological and geometric intricacies of protein structures. Finally, Clustering algorithms are ML techniques used to group similar data points into clusters based on some measure of similarity or distance (Figure 1f). All the structure-based PPI algorithms that will be analyzed in this review are presented in Table 2.

### 3.1. SVM with Radial Basis Function (RBF) Kernel

Bradford et al. developed an SVM method for prediction of protein binding sites using SVMs [52]. The SVM was trained to tell apart interacting and non-interacting surface patches using six surface properties (i.e., surface shape, conservation, electrostatic potential, hydrophobicity, residue interface propensity, solvent accessible surface area (SASA)) as features [52]. The surface shape is defined by two metrics, the shape index (that describes the shape of the local surface) and curvedness, while the conservation score, that was calculated with Scorecons program, was based on sequence homology (BLAST algorithm [63]) and CLUSTALW [52,64]. Next, the electrostatic potential was computed using Delphi software [65,66], incorporating Amber atomic charges [67] and grid-based extrapolation for the protein surface [52]. The Hydrophobicity was calculated using Fauchère and Pliska’s hydrophobicity scale [68]. Finally, the Residue Interface Propensity was calculated from the dataset to indicate whether residues occur more frequently at the interface [52] and SASA was computed using MSMS program [69]. The training dataset was made up of 180 manually curated proteins, representing transient and obligate protein interactions, filtered for natural and stable dimers. To choose the surface patches of the protein, each protein surface was generated using solvent-excluded surfaces (SES) by MSMS. Essentially, an atom is considered part of the interface if >99% of its SASA is eradicated when the protein complex is assembled [52]. Negative samples of equal size as the training dataset and random patches of non-interacting surface regions were also chosen. They also conducted Leave-one-out Cross-Validation (LOOCV) to better evaluate model stability and they thus managed to successfully predict the location of the binding site on 76% of the proteins of the training dataset [52]. Furthermore, it also shows great generalizability across binding types (i.e., obligate and transient binding sites) and potential for functional site discovery, since it can identify functional sites on protein surfaces even when the predicted binding interface does not align with the PDB-specified interface [52]. However, although their patch selection method provides a balanced approach between specificity and sensitivity, there is still room for improvement.

Zhu et al. implemented a two-stage SVM approach (namely, NOXclass) that distinguishes between obligate interactions, non-obligate interactions and crystal packing contacts [53]. The biological difference between the first two interaction types is that protomers of non-obligatory complexes may separate from one another and remain stable and functional components, but protomers of obligate complexes do not exist as stable structures in vivo [53]. Conversely, crystal packing contacts are essentially artifacts of the crystallization process that would not be present in solution or in the physiological state [70]. The developed two-stage SVM first classifies the interaction as biologically relevant or not (i.e., crystal packing interaction), and if and only if the interaction is classified by the SVM1 binary classifier as biologically relevant it is then considered by SVM2 as either an obligate or a non-obligate interaction [53]. NOXclass encompasses a multitude of features that have been used in the literature to differentiate between different protein interaction types (i.e., interface area (IA), ratio of interface area to protein surface area (IAR), amino acid composition of the interface (AAC), correlation between AAC of interface and protein surface (COR), gap volume index (GVI), and conservation score of the interface (CS)) [53]. IA, which represents half the reduction in SASA upon complex formation and IAR, which normalized the interface area by the smaller protomer’s SASA, account for variations in interaction surface sizes and effectively distinguish biological interactions from crystal packing [53]. The AAC evaluated the contribution of different amino acid types at the interface, mainly highlighting differences in hydrophobic and charged residues [53]. To further differentiate biological interfaces, the COR measured how closely the interface AAC matched the overall surface composition of each protein [53]. The GVI quantified shape complementarity by normalizing the gap volume between interacting surfaces of protomers against their interface area [53] (i.e., as the GVI increases, the complementarity of the interacting surfaces is smaller and thus the biological significance of the interaction is likely smaller [71]). Finally, the CS was computed using ConSurf [72] and identified conserved residues, while serving as an indicator of biologically relevant interfaces [53]. The feature selection was conducted using cross-validation accuracy. The best accuracy of 91.8% was achieved when IA, IAR, and AAC were selected, but the accuracy achieved when all features were included was comparable and equal to 89.7% [53]. The addition of the other three features seems to add some noise to the classifier. The training set of the SVM consisted of 243 protein–protein interactions, and more specifically, 75 obligate interactions, 62 non-obligate interactions and 106 crystal packing contacts [53].

The best-performing two-stage SVM was applied to a dataset by Bahadur et al., that included 188 crystal packing contacts, 122 homodimers, and 70 other protein–protein complexes [71]. It achieved 80.0% accuracy in the first stage, lower than its nested cross-validation performance [53], probably due to class imbalance of Bahadur’s dataset [71]. The second stage SVM predicts 84.4% of the homodimers to be obligate, and 78.6% of the remaining complexes to be non-obligate [53], but an accuracy score cannot be calculated since the true labels of the Bahadur dataset are not available. In other words, we do not know what percentage of homodimers or other protein–protein complexes are obligate and what are non-obligate, and thus Zhu et al. assume that the majority of the homodimers are obligate interactions while the majority of other complexes are non-obligate interactions [53].

### 3.2. Random Forest (RF)

The method developed by Maheshwari and Brylinski combines a variety of computational techniques, including molecular modeling, structural bioinformatics, ML, and functional annotation filters [54]. With the use of molecular modeling, docking and docking refinement algorithms, the structures of the proteins in every protein pair are predicted, followed by the identification of their binding site and finally the dimer prediction and its refinement [54]. The ML algorithm of choice is an RF Classifier that is used to predict through assignment of a probability score whether a given dimer represents a true PPI [54]. The features included in the RF classifier of this study are retrieved from the results of FiberDock, a backbone refinement algorithm that calculates attractive and repulsive van de Waals forces, atomic contact energy, partial electrostatics, hydrogen and disulfide bonds, π-stacking, and aliphatic interactions [73]. After the prediction, the protein pairs that were classified as interacting were passed via a GO term filter to ensure they shared cellular locations ([CC]) and participated in the same biological process ([BP]) but had different molecular functions ([MF]) [74]. This unified pipeline utilized the BM1905 dataset previously compilated by the research group, from which two subsets were extracted after processing: 14,944 homodimers (HOM14944) and 3519 heterodimers (HET3519) [54]. The RF model was trained and evaluated using 10-fold cross-validation, employing HET3519 as the positive dataset and RND14944 as the negative dataset [54]. The latter was generated by randomly shuffling the HOM14944 dataset to generate pairs that are not included [54]. Initially, the algorithm was tested on non-interacting protein pairs derived from the Negatome 2.0 database [34]. Subsequently, the model was validated on 6341 known PPIs of *E. coli*, and on 112 interactions of the human immune pathway [54]. The model achieved a Receiver operating characteristic (ROC)-Area Under the Curve (AUC) score of 0.72 for the BM1905 dataset, a false positive rate (FPR) of 0.23 on Negatome 2.0, and 62% accuracy on *E. coli* PPIs [54]. Notably, the model’s discrimination capacity *for E.* *coli* was significantly improved by applying protein localization filters, resulting in an increase in the F-measure from 0.52 to 0.69 [54]. Additionally, the model achieved a predictive accuracy of 62% for the human PPIs in the human immune pathway [54].

Another application of the RF classifier in protein interaction site prediction is the work of Li et al. who combined this ML method that incorporates a variety of single amino acid features ranging from properties of the entire protein, features of the primary and secondary structures of the protein as well as 3D structural features with an intricate feature selection module [55]. The Evolutionary conservation feature was quantified using Position Specific Scoring Matrices (PSSMs) generated by Position-Specific Iterated BLAST (PSI-BLAST) [75], representing the likelihood of each residue to be conserved instead of mutating to each of the 20 amino acids [55]. Amino acid properties were represented using five numerical patterns derived from AAIndex database [76], reflecting polarity, secondary structure, molecular volume, codon diversity, and electrostatic charge [55]. Protein-disordered regions, crucial for biological functions and interaction versatility, were analyzed in this study using VSL2 [77] to calculate disorder scores for each amino acid [55]. Secondary structural features, including secondary structure (labels: ‘helix’, ‘strand’, or ‘other’) and solvent accessibility (labels: ‘buried’ (excluded from the study) or ‘exposed’) were predicted by SSpro4 [78]. Finally, the 3D structural features extracted from PDB database that were used in this study were Protrusion Index (CX) and Depth Index (DPX) predicted by PSAIA [79], as well as SASA, molecular surface area (MS) and surface curvature (SC) computed from SurfRace [80]. The Minimum Redundancy Maximal Relevance (mRMR) method was employed to rank features by evaluating their relevance to the target and their redundancy with other features [81]. The resulting ranked list was then used in the Incremental Feature Selection (IFS) [82,83] process to determine the optimal feature set. The PPI training dataset was constructed from the 3did database [84], focusing on interactions with known structures. After filtering out short sequences and homologs [85], 6488 protein chains from 3353 PDB structures were retained [55]. From these, 21-residue segments centered on interaction residues were extracted and thus positive samples (interaction residues) and negative samples (non-interaction residues) were identified, resulting in 104,802 positive and 180,698 negative samples [55]. After excluding peptides centered on buried residues and further reducing homology [85], the dataset was refined to 13,427 positive and 12,429 negative samples [55]. Interestingly, the study demonstrated that 3D structural features significantly improve PPI site prediction, resulting in a prediction accuracy of 67.3% and a Matthews correlation coefficient (MCC) of 0.348 compared to 59.7% accuracy and an MCC of 0.190 without these features [55]. A comparison with the method proposed by Šikić et al. [86] on their same dataset further validated the approach [55]. Using the RF and 10-fold cross-validation for all experiments, the proposed method achieved better performance (accuracy: 67.3%, MCC: 0.348) than the method Šikić et al. [86] (accuracy: 65.3%, MCC: 0.308) [55].

### 3.3. Bayesian Network

PrePPI method leverages structural and non-structural features to model PPIs across yeast and human proteomes [56]. At first, 6521 yeast proteins were matched to 7792 domains, while 20,318 human proteins were matched to 49,851 domains using SMART [87]. Structures were sourced directly from PDB for high-sequence-identity matches or derived from ModBase [88] and SkyBase [89] homology models in cases where proteins did not have experimentally verified structures [56]. The selection process resulted in a total of 1361 PDB structures and 7222 homology models for yeast proteins, and 8582 PDB structures and 30,912 models for human proteins [56]. Structural neighbors (both close and remote) were identified using the Ska alignment tool that performs structural alignment depending only on the geometric shape of the two proteins [56]. If two of the neighbors of the two proteins in every protein pair are found in a complex formation in PDB and PQS [56] (28,408 yeast and 29,012 human protein complexes), their complex serves as a potential template to create models of the interaction between the proteins in question [56]. The “models” of the complex come from superimposing the structures of the proteins in question to their neighbors that create the template [56]. This strategy generated over 550 million “models” (2.4 million PPIs) for 3900 yeast proteins, and 12 billion models (36 million PPIs) for 3000 human proteins [56]. Afterward, five structural modeling features were calculated to evaluate the created “models”. Structural Similarity (SIM), Size of Conserved Interacting Pairs (SIZ) and Coverage of Interacting Pairs (COV) show whether the interface of a template is present in the “model”, and finally Overlap Score (OS) and Overlap of Predicted Interfacial Residues (OL) evaluate if the residues in the “model” interface have compatible characteristics with residues that mediate recognized PPIs [56]. All the features mentioned above were combined into a Likelihood score using a Bayesian Network [56]. This score was then incorporated with different types of non-structural information, such as gene co-expression, protein essentiality, GO term similarity, and phylogenetic profiles—using a Bayesian classifier to assign interaction likelihood ratios [56]. A naive Bayes classifier was trained on high-confidence and non-interaction reference sets using tenfold cross-validation to evaluate prediction performance through Overlap of Predicted Interfacial Residue curves [56]. This integrative pipeline highlights the power of combining structural modeling with diverse biological data to predict PPIs at scale. Recently, they have updated their PrePPI database (available at: https://honiglab.c2b2.columbia.edu/PrePPI/(accessed on 1/12/2024)), a webserver that predicts PPIs on a proteome-wide scale [90].

### 3.4. Artificial Neural Networks (ANNs)

Fariselli et al. applied a neural network-based technique to predict protein interaction sites of heterodimers from 3D structural features [57]. More specifically, it predicts whether each surface residue is in contact with the other protein using an 11-residue window that includes the residue of interest and its spatial neighbors [57]. Features extracted for this prediction include evolutionary conservation profiles derived from sequence alignments in the HSSP database [91] and solvent accessibility that is calculated by DSSP program, with residues coded into 20-dimensional vectors representing their sequence conservation frequencies [57]. The training dataset includes heterodimers, while excluding homodimers and protease–inhibitor complexes, that have specific motifs in their interaction sites, as well as small fragments, resulting in 226 interacting protein chains [57]. A three-fold cross-validation process was employed for validation, achieving a 73% per-residue accuracy and subsequently, the model was also tested on the DnaK molecular chaperone system, showing strong agreement with known experimental interaction regions [57].

### 3.5. Convolutional Neural Networks (CNNs)

A recent structure-based method for PPI prediction is SpatialPPI, which integrates protein complexes predicted using AlphaFold Multimer and classifies their interactions through 3D CNNs [58]. Rigorous curation processes were implemented to address class imbalance and eliminate redundancy, as a considerable overlap exists between protein pairs in both data sources (i.e., BioGRID and Negatome 2.0) [58]. The model was thus trained on a dataset comprising of 600 positive PPI pairs from the BioGRID database [43] and 600 non-interactive pairs curated from Negatome 2.0 [43]. For each protein pair, amino acid sequences were input into AlphaFold Multimer [92], which predicted individual protein structures and optimized the resulting complex. Subsequent steps involved feature extraction from the predicted protein complex, converting the structure into a 3D tensor to represent spatial atomic arrangements [58]. Three encoding strategies were employed for this purpose: one-hot, volume, and distance encoding [58]. These encoded features were then processed by the 3D CNN, followed by fully connected layers, which ultimately classified the protein complex as either interacting (PPI) or non-interacting (non-PPI) [58]. Two distinct CNN architectures were explored: one utilizing Residual blocks and the other employing Dense blocks, which are 3D adaptations of the ResNet [93] and DenseNet [94] frameworks, respectively. Both architectures incorporated convolutional layers augmented by dropout, batch normalization to enhance generalization and stability, as well as average 3D pooling to calculate 1D feature vector [58]. Validation of the SpatialPPI model was performed using five-fold cross-validation, coupled with a clustering-based subset selection strategy to ensure that similar proteins were assigned to the same subset [58]. Multiple combinations of the CNN architectures and tensorization methods were initially evaluated, with the DenseNet-based 3D CNN utilizing distance tensorization emerging as the most accurate. This optimal configuration achieved a mean accuracy of 0.81, an AUC score of 0.89, a precision of 0.83, and a recall of 0.79, while maintaining low average and standard deviation of accuracy across folds, highlighting its stability [58]. Furthermore, SpatialPPI demonstrated superior performance compared to other state-of-the-art methods, including sequence-based approaches, as evidenced by its results on the test subset employed in DeepTrio [22]. SpatialPPI model achieved 0.83 accuracy, 0.92 AUC score, 0.84 precision and 0.82 recall. Finally, evaluation on the CASP14 dataset [95] showed that SpatialPPI produced fewer false predictions when compared to docking-based methods, reinforcing its robustness and reliability.

### 3.6. Graph Neural Networks (GNNs)

The Struct2Graph model employs a graph attention network (GAT) for PPI prediction, using only a graph representation of 3D structural data of proteins, and not specific structural features, such as SASA and hydrophobicity, that have been employed by other researchers [26]. The training dataset includes 117,933 protein pairs (4698 positive and 5036 negative pairs) with available structures from PBD database [37]. On one hand, the positive pairs are derived from concordant matches between physical PPIs of STRING [45] and IntAct [44], after excluding co-localized proteins [26]. On the other hand, negative samples are considered to be any protein pairs that show no interaction evidence in large-scale two-hybrid studies and are also not part of any interaction in either STRING or IntAct [26]. The model framework converts protein structures into graphs where amino acids are represented as nodes, connected by edges if their spatial proximity is within 9.5 Å [26]. Local structural information is captured via 1-neighborhood subgraphs [96], which are then processed by graph convolutional networks (GCNs) [26]. The resulting protein embeddings are aggregated using a mutual attention mechanism, and the final classification is performed with a feedforward neural network [26]. Model evaluation was conducted using five-fold cross-validation on both balanced datasets and unbalanced scenarios with varying positive-to-negative ratios [26]. On the balanced dataset, the model achieved outstanding performance with an average accuracy of 98.96%, precision of 99.4%, recall of 98.57%, and F1-score of 98.98% [26]. Even under class-imbalance conditions, the model maintained robust metrics, demonstrating its generalization capability [26]. For instance, in the most challenging scenario with a 1:10 positive-to-negative ratio, the model achieved 99.26% accuracy, 97.04% precision, 95.59% recall, and 96.31% F1-score [26]. These results highlight the exceptional generalization ability of the Struct2Graph model, making it suitable not only for supervised learning tasks but also for unsupervised applications [26]. Lastly, the attention maps generated by the model provide valuable insights for identifying potential interaction-critical residues [26].

The study by Jha et al. introduces a method for predicting PPIs by combining GNNs with language models to create enhanced structural protein representations [59]. The training dataset includes 16,220 positive human PPIs and 2847 positive PPIs from *Saccharomyces cerevisiae* (*S. cerevisiae*) from Human Protein Reference Database (HPRD) [97] and DIP [98], correspondingly. Negative samples were constructed by randomly pairing proteins from the positive dataset that are localized in different subcellular locations (as annotated in Swiss-Prot [99]) and supplementing these with non-PPIs from the Negatome database [34], resulting in 5997 negative human and 4427 negative *S. cerevisiae* interactions. Further filtering steps were applied to the dataset, which involved excluding short protein chains, removing protein pairs with ≥40% sequence identity (using CD-HIT [85]), and discarding proteins lacking 3D structures in the PDB database [26]. The features were extracted using two methods derived from language models, i.e., SeqVec that employs Long Short-Term Memory (LSTM) layers [100], and ProtBert that extends the Bidirectional Encoder Representations from Transformers (BERT) framework [101]. These language models were adapted to extract residue-level features, which were subsequently used as node features in the protein structure-based graphs [59]. After the extraction, both GATs and GCNs were evaluated for generation of protein embeddings [59]. These embeddings were then concatenated, and the final fully connected network layer performed the prediction [59]. The framework was comprehensively evaluated to determine the optimal combination of GNN architecture, language model, and other node features (e.g., one-hot encoding and physicochemical properties) [59]. The evaluation also included assessing the impact of varying GNN layers, dataset sample sizes, and comparisons with language model-based baselines and previous methodologies [59]. The selected model was further validated using five-fold cross-validation for stability and tested on human and *S. cerevisiae* datasets [59]. The optimal model—a GAT architecture combined with the LSTM-based SeqVec for node features—achieved exceptional results, with an accuracy of 98.13% on the human dataset and 92.15% on the *S. cerevisiae* dataset [59].

Microenvironment-Aware Protein Embedding for PPI prediction (MAPE-PPI) [60], a computational technique utilizing microenvironment-aware protein embeddings to predict large-scale PPI types using Graph Isomorphism Networks (GINs) [102]. MAPE-PPI extracts microenvironment-aware embeddings based on the protein’s structure, captured in a fine-grained “codebook” through a variant of vector-quantized variational autoencoders (VQ-VAE) [103]. Essentially, this microenvironment codebook encodes each residue into chemically meaningful discrete codes, that contain information relevant to each residue’s surrounding chemistry and geometry [60]. The final calculated protein embedding serves as node features in a PPI graph that is made up of all the proteins whose interactions are to be predicted (i.e., hidden edge types) and the PPIs of the training dataset [60]. Next, the GIN architecture serves as an encoder and the final classification is implemented by a fully connected layer that categorizes the interaction to at least one of the following seven interaction types, i.e., Activation, Binding, Catalysis, Expression, Inhibition, Post-translational modification, and Reaction [60]. There are three training datasets, one for each of the conducted computational experiments [60]. The first one includes PPIs from STRING (1,150,830 PPIs, 14,952 proteins) and the other two are two subsets of STRING, namely SHS27k (16,912 PPIs), and SHS148k (99,782 PPIs) [45], that contain human PPIs. All the protein structures were derived from AlphaFold2 database [39]. For each training dataset, three splitting techniques were evaluated: Random Split, Breath-First Search (BFS), and Depth-First Search (DFS) [60]. The computational experiment that uses STRING as the training dataset achieved 96.12 micro-F1-score when the model was trained from scratch and 96.9 micro-F1-score when using pre-training data [60].

The Hierarchical Graph Neural Network for Protein–Protein Interactions (HIGH-PPI) [61] is an advanced framework designed for multi-type PPI prediction, integrating a hierarchical graph structure and an explainer module to identify key residues involved in protein interactions. This hierarchical representation encapsulates both residue-level and protein-level interactions, enabling the model to learn fine-grained and global features simultaneously [61]. In this architecture, individual proteins are represented as residue-level subgraphs, where nodes correspond to residues and edges represent spatial or sequential proximity, while PPIs are modeled as higher-level nodes in a global graph [61]. This dual-layer structure facilitates the simultaneous capture of intra-protein and inter-protein interactions. The dataset utilized for developing HIGH-PPI is derived from SHS27k, a curated subset of human PPIs extracted from the STRING database [45]. To ensure structural consistency, PPIs involving proteins without resolved structures in the PDB were excluded [37], resulting in a final dataset comprising approximately 1600 proteins and 6600 PPIs [61]. The model employs a dual-view approach: a bottom view representing proteins as residue-level graphs and a top view modeling the protein interaction network [61]. The bottom view utilizes GCNs to extract residue-level features, such as isoelectric point, polarity, acidity/alkalinity, hydrogen bond properties, octanol-water partition coefficient, and topological polar surface area [61]. Protein graphs are constructed using a distance cutoff of 10 Å for adjacency [61]. The top view employs GINs to capture network-level PPI properties [61]. The embeddings from the bottom view are used as inputs for the PPI graph processed by the top view, and the resulting protein embeddings are subsequently classified using a Multi-Layer Perceptron (MLP) [61]. In addition to classification, HIGH-PPI incorporates an Explainable Artificial Intelligence (XAI) component, specifically GNNExplainer [104], to identify functionally critical residues such as binding or catalytic sites [61]. Evaluation of HIGH-PPI demonstrates its superior performance, robustness, and generalization capabilities compared to state-of-the-art methods [61]. The model was tested using a random data split, with 20% of PPIs reserved for evaluation. HIGH-PPI achieved the highest micro-F1-score, surpassing the second-best model by approximately 4% and attaining a micro-F1-score of ~90% [61]. Robustness testing under random perturbations revealed a performance improvement of up to 19% compared to the next-best baseline, highlighting the model’s resilience to noisy data [61]. Generalization assessments in out-of-distribution (OOD) scenarios further confirmed HIGH-PPI’s consistent superiority over alternative methods [61]. Moreover, HIGH-PPI demonstrated significant improvements in area under the precision–recall curve (AUPR) across five PPI types, with particularly strong performance in binding-type predictions [61]. The robustness and generalizability of HIGH-PPI were further validated on additional datasets, including proteins with computationally predicted structures, such as those generated by AlphaFold [39], and real catalytic site annotations [61]. Across these datasets, HIGH-PPI exhibited consistent and robust performance, reinforcing its applicability and effectiveness in diverse PPI prediction tasks [61].

### 3.7. Clustering

The AlphaBridge framework, a recent advancement in the computational analysis of protein complexes, leverages cutting-edge metrics from AlphaFold3 [105], including the predicted local-distance difference test (pLDDT), pairwise aligned error (PAE), and predicted distance error (PDE) [62]. Integrating these metrics into a graph-based clustering approach, enables the precise identification and analysis of interaction interfaces in macromolecular complexes, including protein–protein and protein–nucleic acid interactions [62]. Interaction data are visualized through sophisticated chord diagrams, incorporating prediction confidence and sequence conservation scores to enhance interpretability. The framework utilizes structural metrics derived from AlphaFold3 outputs, with preprocessing centered on constructing the Predicted Merged Confidence (PMC) matrix—a fusion of PAE and pLDDT data—further refined through community clustering algorithms and multidimensional image processing techniques [62]. Empirical evaluation involved AlphaFold3-predicted models, including cases such as human mismatch repair proteins interacting with nucleic acids [62]. Validation focused on the robustness of predictions, employing interactive visualizations to assess confidence levels and identify physiologically relevant interactions [62].

## 4. Conclusions

Structure-based PPI prediction represents a far more accurate framework than sequence-based methods due to its capacity of capturing the spatial and biochemical complexities of protein interactions. Protein structures provide invaluable insights into the three-dimensional arrangements of residues, information that is critical for understanding binding sites, catalytic mechanisms, and overall interaction dynamics. They contain features such as atomic coordinates, solvent accessibility, and interaction geometry, that allow for a deeper exploration of the molecular mechanisms underpinning PPIs, often missed by sequence-centric methods. For instance, sequence-based models may fail to differentiate conformations of the same protein that bind different partners, as structural variability cannot be inferred directly from sequences alone. However, challenges such as the reliance on high-resolution structural data and the computational expense of three-dimensional modeling underline the need for further innovation to make these methods more scalable and applicable across diverse proteomes.

Another crucial aspect of structure-based methods is the integration of reliable negative samples, which remains a critical challenge in PPI prediction. Experimental identification of non-interacting protein pairs is practically infeasible, since biological experiments are designed to determine protein interactions and not the opposite. The main approaches for this issue are the random generation of “negative” interactions from experimentally verified positive interactions, or the use of databases containing “negative” interactions, like Negatome 2.0. However, in some cases, “negative” interactions from these databases were spotted in protein interaction databases, indicating that they probably should not be trusted blindly. In some other cases, randomly generated negative interactions are also filtered so that the proteins that make the pair belong in different subcellular locations or organs. Recent studies have proposed the use of “negative” interactions of proteins from high-throughput experiments, which are essentially interactions between proteins that although tested experimentally, were not observed [35]. However, these strategies can introduce biases, overestimate prediction accuracy, or create imbalances in datasets, particularly in highly specific biological contexts. The selection of meaningful negative samples, while complex, will play a decisive role in improving the robustness and reliability of predictive models. Another issue concerning the training process of the ML algorithms is the analogy between the positive and negative datasets. In most cases, the chosen training dataset is balanced, but this approach is rather simplistic because two proteins most likely do not interact. Consequently, this problem should be addressed by using imbalanced training datasets with the majority class being the “negative” interactions accompanied with the use of functions that help mitigate the imbalance, like the Focal Loss function. Additionally, this problem could also be solved as a weakly supervised learning problem, since the “negative” interaction data are often ambiguous.

The comparison of PPI prediction methods reveals diverse approaches with varying strengths and limitations. On one hand, SVMs excel in handling small, high-dimensional datasets and feature-rich inputs, such as those derived from chemical descriptions. However, they often struggle with scalability and require careful feature selection to avoid overfitting. RFs, on the other hand, are robust to noisy data and capable of handling large feature sets, yet not only can their interpretability be challenging but they also require significant computational resources for large-scale datasets. Deep models, such as ANNs and CNNs, offer superior capabilities in learning intricate patterns, especially when applied to spatial and structural features. However, these models demand extensive computational resources, large and annotated datasets, as well as careful tuning of hyperparameters to achieve optimal performance. Furthermore, while CNNs have shown great promise for spatial pattern recognition, their capacity to capture the full 3D protein structure remains limited compared to more specialized models. Graph-based approaches like GNNs have emerged as state-of-the-art methods due to their ability to directly model protein structures as graphs, capturing complex topologies inherent in biological interactions. GNNs effectively handle relational data and can model PPIs with remarkable precision. However, these models can be sensitive to graph construction parameters and are computationally expensive when dealing with large protein interaction networks. Moreover, graph transformers, an advanced variant, have begun to exploit the potential of 3D protein structures, providing a promising avenue for more accurate PPI predictions by leveraging the spatial properties of proteins. These models are better equipped to handle the complexities of protein folding and spatial interactions, but they still require significant resources to process large-scale, high-dimensional datasets. While each method has demonstrated utility in specific applications, no single approach universally excels across all scenarios, emphasizing the need for tailored strategies based on the data and prediction goals. This highlights the growing importance of hybrid models that combine strengths from different approaches to achieve superior performance, particularly in complex biological systems where data can be both sparse and highly dimensional.

To overcome the limitations of the existing PPI prediction methods, targeted advancements are necessary. For SVMs, incorporating automated feature engineering, such as DL-based feature extraction, could enhance their ability to handle large-scale datasets without manual preprocessing. In the case of RFs, improving their interpretability through feature importance visualization or integrating explainable AI techniques can make them more accessible for biological insights. For ANNs and CNNs, transfer learning using pre-trained models on large PPI datasets could mitigate the challenge of limited annotated data, especially in non-interaction scenarios. These models could also benefit from incorporating domain-specific constraints to reduce overfitting and integrating knowledge of protein structures to enhance their performance in structural-based prediction tasks. Graph-based approaches would benefit from adaptive graph construction techniques that dynamically adjust graph topology based on biological context, along with hybrid models that combine graph-based insights with other structural and sequence-based features. Additionally, leveraging the full potential of 3D protein structures through graph transformers and other DL architectures designed to exploit the spatial properties of proteins could lead to more precise and scalable PPI predictions. Furthermore, integrating multi-omics data and functional annotations into these frameworks could enrich predictions and expand their utility for uncovering novel interactions in complex biological systems, making them more versatile and accurate for a wide range of applications in drug discovery and disease understanding.

The integration of XAI can provide a path for non-expert users to trust computational tools in PPI prediction [106]. XAI models, which enhance transparency by elucidating the reasoning behind predictions, can be incorporated into structure-based prediction methods to identify the specific features or data driving model outputs [107]. For example, visualization tools can highlight crucial structural motifs or residues contributing to a predicted interaction, enabling users to assess the reliability of results. Additionally, tools like SHapley Additive exPlanations (SHAP) [108] or Local Interpretable Model-Agnostic Explanations (LIME) [109] can help quantify the confidence of predictions by showing the importance of individual input features. These insights empower users to contextualize results within biological frameworks, promoting informed decision-making. By making these tools intuitive and embedding user-friendly confidence metrics, researchers can bridge the knowledge gap, enabling broader adoption of these computational methods while maintaining reliability and reproducibility in experimental applications.

The trajectory of computational PPI tools suggests a future where their predictions achieve near-experimental reliability, fundamentally transforming biological research. Neural network-based methods are rapidly improving in accuracy due to advancements in data availability, structure prediction models (e.g., AlphaFold), and algorithmic efficiency. These tools are particularly impactful in drug discovery and testing, where they offer a viable alternative to animal models. By accurately predicting PPIs, computational methods can simulate the effects of potential drug candidates on molecular pathways, enabling the identification of therapeutic targets and adverse interactions in silico. For instance, in silico studies of protein–ligand docking combined with PPI predictions were used to identify inhibitors for diseases such as cancer, bypassing early-stage animal testing [107]. This reduces the ethical concerns associated with animal models and ensures a faster, cost-effective path to drug validation. Moreover, PPI predictions are integral to advancements in personalized medicine. By understanding the precise interactions within an individual’s proteome, researchers can tailor treatments to target specific pathways affected by disease, exemplified by cancer therapies that inhibit specific protein interactions unique to tumor biology. The ethical exploration of biology also benefits from such computational tools, as they minimize experimental redundancies, promote data sharing, and align research with frameworks advocating humane and efficient methodologies [110].

In conclusion, structure-based PPI prediction stands as a pivotal tool in modern computational biology. It offers profound insights into the molecular mechanisms governing protein interactions while overcoming critical limitations of traditional methodologies. Future research should focus on improving the scalability of these approaches, optimizing the generation of negative datasets, and expanding their application to proteomes with limited structural data. By addressing these challenges, structure-based methods have the potential to redefine our understanding of protein networks and their implications in health and disease.

## Figures and Tables

**Table 1:** Most-used protein interaction and protein feature databases.

	Databases	Description	URL	Last Update	References
Primary PPI Databases	DIP	Experimentally curated PPI database that also includes biological information of proteins.	https://dip.doe-mbi.ucla.edu/dip/Main.cgi (accessed on 01/12/2024)	2020	[41]
	MINT	Experimentally curated PPI database that contains about 235,635 non-redundant interaction pairs from 4786 manually curated publications.	https://mint.bio.uniroma2.it/ (accessed on 01/12/2024)	2012	[42]
	Biogrid	Manually curated PPI data from 85,855 publications, i.e., 2,818,695 protein and genetic interactions, 31,144 chemical interactions and 1,128,339 post translational modifications.	http://www.thebiogrid.org (accessed on 01/12/2024)	2024	[43]
	IntAct	Curated resource of molecular interactions, both from the scientific literature and direct data depositions containing 1,624,377 binary interactions.	http://www.ebi.ac.uk/intact (accessed on 01/12/2024)	2024	[44]
PPI Meta-databases	STRING	Functional associations between protein pairs that covers 12,535 organisms, 59,309,604 proteins and a total of 27,541,372,832 PPIs of various confidence levels.	https://string-db.org/ (accessed on 01/12/2024)	2023	[45]
	Mentha	A comprehensive resource that integrates 741,337 PPIs from several primary databases such as IntAct, BioGRID, and others.	http://mentha.uniroma2.it (accessed on 01/12/2024)	2024	[46]
	IMEx Consortium (International Molecular Exchange Consortium)	Aggregation of more than 1.5 million data from databases like IntAct, MINT, and DIP to provide standardized and non-redundant PPI data.	https://www.imexconsortium.org (accessed on 01/12/2024)	2024	[47]
	iRefIndex (Integrated Reference Interactome)	Meta resource based on matching protein sequence data, providing access to a large collection of protein–protein interaction data.	https://irefindex.vib.be (accessed on 01/12/2024)	2023	[48]
	HINT	Curated collection of high-quality protein–protein interactions from 8 interactome resources.	https://hint.yulab.org/ (accessed on 01/12/2024)	2024	[49]
	OmniPath	Meta-database that combines data from more than 100 resources and besides PPIs, it also contains gene regulatory interactions, enzyme-post-translational modifications relationships, protein complexes, protein annotations and intercellular communication information.	https://omnipathdb.org/ (accessed on 01/12/2024)	2020	[50]
	PICKLE	Meta-database for the direct protein–protein interactome of the human and the mouse proteomes.	http://www.pickle.gr/ (accessed on 01/12/2024)	2021	[51]
Protein Non-Interaction Databases	Negatome Database 2.0	Database of proteins and protein domains that are unlikely to engage in physical interactions based on manual curation of the scientific literature.	https://mips.helmholtz-muenchen.de/proj/ppi/negatome/ (accessed on 01/12/2024)	2014	[34]
Protein Features	UniProt	A collection of 248,838,887 protein sequences annotated with functional information.	http://www.uniprot.org (accessed on 01/12/2024)	2024	[40]
	PDB	Experimentally determined 3D structures of proteins	http://www.rcsb.org/ (accessed on 01/12/2024)	2024	[36,37]
	AlphaFold Database	Extensive database of 200 million high-accuracy protein-structure predictions.	https://alphafold.ebi.ac.uk/ (accessed on 01/12/2024)	2024	[38,39]

**Table 2:** Structure-based PPI prediction algorithms.

Computational Methodology	Title	Doi	Authors	Year	Citation
SVM with radial basis function (RBF) kernel	Improved prediction of protein–protein binding sites using a support vector machines approach	https://doi.org/10.1093/bioinformatics/bti242	Bradford and Westhead	2005	[52]
	NOXclass: prediction of protein–protein interaction types	https://doi.org/10.1186/1471-2105-7-27	Zhu et al.	2006	[53]
Random Forest (RF)	Across-proteome modeling of dimer structures for the bottom-up assembly of protein–protein interaction networks	https://doi.org/10.1186/s12859-017-1675-z	Maheshwari and Brylinski	2017	[54]
	Prediction of Protein–Protein Interaction Sites by Random Forest Algorithm with mRMR and IFS	https://doi.org/10.1371/journal.pone.0043927	Li et al.	2012	[55]
Bayesian Networks	Structure-based prediction of protein–protein interactions on a genome-wide scale	https://doi.org/10.1038/nature11503	Zhang et al.	2013	[56]
Artificial Neural Networks (ANNs)	Prediction of protein–protein interaction sites in heterocomplexes with neural networks	https://doi.org/10.1046/j.1432-1033.2002.02767.x	Fariselli et al.	2002	[57]
Convolutional Neural Network (CNNs)	SpatialPPI: Three-dimensional space protein–protein interaction prediction with AlphaFold Multimer	https://doi.org/10.1016/j.csbj.2024.03.009	Hu and Ohue	2024	[58]
Graph Neural Networks (GNNs)	Struct2Graph: a graph attention network for structure-based predictions of protein–protein interactions	https://doi.org/10.1186/s12859-022-04910-9	Baranwal et al.	2022	[26]
	Prediction of protein–protein interaction using graph neural networks	https://doi.org/10.1038/s41598-022-12201-9	Jha et al.	2022	[59]
	MAPE-PPI: Towards Effective and Efficient Protein–Protein Interaction Prediction via Microenvironment-Aware Protein Embedding	https://doi.org/10.48550/arXiv.2402.14391	Wu et al.	2024	[60]
	Hierarchical graph learning for protein–protein interaction	https://doi.org/10.1038/s41467-023-36736-1	Gao et al.	2023	[61]
Clustering	AlphaBridge: tools for the analysis of predicted macromolecular complexes	https://doi.org/10.1101/2024.10.23.619601	Álvarez-Salmoral et al.	2024	[62]

## Data Availability

This article does not contain any original data. The content is based entirely on previously published literature, which is cited throughout the manuscript.

## Abbreviations

PPI: Protein–Protein Interaction; ML: Machine Learning; DL: Deep Learning; FP: Fluorescence polarization; SPR: Surface plasmon resonance; NMR: Nuclear magnetic resonance; CD: Circular dichroism; SLS/DLS: Static and dynamic light scattering; ITC: Isothermal titration calorimetry; FRET: Fluorescence resonance energy transfer; BRET: Bioluminescence resonance energy transfer; PCA: Protein-fragment complementation assay; MS: Mass spectrometry; Y2H: Yeast two-hybrid; DDI: domain–domain interaction; RF: Random Forest; AI: Artificial intelligence; SVM: Support Vector Machine; MLD: Multi-scale local descriptor; GBDT: Gradient Boosting Decision Tree; E-ELM: Ensemble Extreme Learning Machine; PCA: Principal Component Analysis; AC: Autocovariance; CT: Conjoint Triad; SAE: Stacked Autoencoder; CNN: Convolutional Neural Network; PDB: Protein Data Bank; GO: Gene Ontology; ANNs: Artificial Neural Networks; GNN; Graph Neural Network; SASA: Solvent accessible surface area; SES: Solvent excluded surfaces; LOOCV: Leave-one-out Cross-Validation; IA: Interface area; IAR: Ratio of interface area to protein surface area; AAC: Amino acid composition of the interface; COR: Correlation between AAC of interface and protein surface; GVI: Gap volume index; CS: Conservation score of the interface; CC: Cellular location; BP: Biological process; MF: Molecular function; ROC: Receiver operating characteristic; AUC: Area under the curve; FPR: False positive rate; PSSM: Position Specific Scoring Matrices; PSI-BLAST: Position-Specific Iterated BLAST; CX: Protrusion Index; DPX: Depth Index; MS: Surface area; SC: Surface curvature; mRMR: Minimum redundancy maximal relevance; IFS: Incremental Feature Selection; MCC: Matthews correlation coefficient; SIM: Structural similarity; SIZ: Size of conserved interacting pairs; COV: Coverage of Interacting Pairs; OS: Overlap score; OL: Overlap of predicted interfacial residues; GAT: Graph attention network; GCN: Graph convolutional network; HPRD: Human Protein Reference Database; LSTM: Long Short-Term Memory; BERT: Bidirectional Encoder Representations from Transformers; MAPE-PPI: Microenvironment-Aware Protein Embedding for PPI prediction; GIN: Graph Isomorphism Network; VQ-VAE: Vector-quantized variational autoencoders; BFS: Breath-First Search; DFS: Depth-First Search; HIGH-PPI: Hierarchical Graph Neural Network for Protein–Protein Interactions; MLP: Multi-Layer Perceptron; XAI: Explainable artificial intelligence; OOD: Out-of-distribution; AUPR: area under the precision–recall curve; pLDDT: Predicted local-distance difference test; PAE: Pairwise aligned error; PDE: Predicted distance error; PMC: Predicted merged confidence; SHAP, SHapley Additive exPlanations; LIME, Local Interpretable Model-Agnostic Explanations.
